# Jujuboside B Reduces Vascular Tension by Increasing Ca^2+^ Influx and Activating Endothelial Nitric Oxide Synthase

**DOI:** 10.1371/journal.pone.0149386

**Published:** 2016-02-22

**Authors:** Yixiu Zhao, Xin Zhang, Jiannan Li, Yu Bian, Miaomiao Sheng, Bin Liu, Zidong Fu, Yan Zhang, Baofeng Yang

**Affiliations:** 1 Department of Pharmacology, Harbin Medical University, Harbin, Heilongjiang, PR China; 2 Key Laboratory of Cardiovascular Medicine Research (Harbin Medical University), Ministry of Education, Harbin, Heilongjiang, PR China; Emory University, UNITED STATES

## Abstract

Jujuboside B has been reported to have protective effect on many cardiovascular diseases. However, the effects of Jujuboside B on vascular tension and endothelial function are unknown. The present study investigated the effects of Jujuboside B on reducing vascular tension, protecting endothelial function and the potential mechanisms. The tension of isolated rat thoracic aorta ring was measured by Wire myograph system. The concentration of nitric oxide (NO) and the activity of endothelial nitric oxide synthase (eNOS) in human aortic endothelial cells (HAECs) were determined by Griess reagent method and enzyme-linked immune sorbent assay. The protein levels of eNOS and p-eNOS at Serine-1177 were determined by western blot analysis. Intracellular Ca^2+^ concentration in HAECs was measured by laser confocal imaging microscopy. Results showed that Jujuboside B reduced the tension of rat thoracic aorta rings with intact endothelium in a dose-dependent manner. L-NAME, KN93, EGTA, SKF96365, iberiotoxin and glibenclamide significantly attenuated Jujuboside B-induced vasodilation in endothelium-intact tissues. In contrast, indometacin and 4-DAMP had no such effects. Jujuboside B also promoted NO generation and increased eNOS activity, which were attenuated by L-NAME, EGTA and SKF96365. Moreover, Jujuboside B increased intracellular Ca^2+^ concentration dose-dependently, which was inhibited by EGTA and SKF96365. Besides, Jujuboside B induced a rapid Ca^2+^ influx instantaneously after depleting intracellular Ca^2+^ store, which was significantly inhibited by SKF96365. In conclusion, this study preliminarily confirmed that Jujuboside B reduced vascular tension endothelium-dependently. The underlying mechanisms involved that Jujuboside B increased extracellular Ca^2+^ influx through endothelial transient receptor potential cation (TRPC) channels, phosphorylated eNOS and promoted NO generation in vascular endothelial cells. In addition, Jujuboside B-induced vasodilation involved endothelium-dependent hyperpolarizaiton through endothelial potassium channels. Jujuboside B is a natural compound with new pharmacological effects on improving endothelial dysfunction and treating vascular diseases.

## Introduction

Vascular diseases, including atherosclerosis, thrombus and vascular inflammation, have become worldwide epidemics in modern society. Vascular diseases affect lumen caliber and induce ischemia, hypoxia and necrosis of tissues and organs, such as acute myocardial ischemia, cerebral infarction and hypertension [1]. Vascular endothelium secretes multiple factors to modulate vascular tension, platelet activity and thrombogenicity. These factors also affect migration, proliferation of vascular cells and inflammation, atherosclerosis of vasculature in the long term [2]. Vascular endothelial cells (VECs) produce endothelium-derived relaxing factors (EDRFs) and endothelium-derived contracting factors (EDCFs) to relax or contract blood vessels. The balance between EDRFs and EDCFs is essential to maintain vascular tension and endothelial function [3]. However, in disease status, such as hypertension, abnormal hemodynamic signals disturb the balance between EDRFs and EDCFs, trigger preternatural vasoconstriction and induce endothelial dysfunction [4]. Therefore, promoting generation of EDRFs or reducing generation of EDCFs makes for inhibiting abnormal vasoconstriction and preventing endothelial dysfunction.

Sheer stress, hypoxia and vasoactive neurotransmitters in blood are physiological signals for VECs to release EDRFs [5]. EDRFs include multiple vasoactive factors, such as nitric oxide (NO), prostacyclin (PGI_2_) and endothelium-derived hyperpolarizing factors (EDHFs). NO is the most significant and representative EDRF [6]. Endothelial nitric oxide synthase (eNOS) produces NO in response to various stimuli. NO activates guanylate cyclase and convers guanosine triphosphate to cyclic guanosine monophophate (cGMP) in vascular smooth muscle cells (VSMCs). cGMP modulates protein kinase G and induces vasodilation consequently [7]. In addition to NO, PGI_2_ releases from VECs, binds to TP receptors on the VSMC membrane, activates adenylyl cyclase and protein kinase A (PKA) signal transduction pathway and induces vasodilation [8]. Endothelial NO and PGI_2_ also participate in regulating vascular homeostasis and platelet aggregation [9]. In this process, eNOS and phospolipase A2, the key enzymes generating NO and PGI_2_, are classified as Ca^2+^/Calmodulin (CaM)-dependent enzymes which can be activated by various agonists increasing intracellular Ca^2+^ concentration. Intracellular Ca^2+^ results mainly from extracellular Ca^2+^ influxing through calcium channels on VECs membrane and Ca^2+^ releasing from intracellular endoplasmic reticulum Ca^2+^ store through exciting relevant receptors [10]. Ca^2+^/CaM complex activates eNOS by activating CaM kinase II and phosphorylating eNOS at Serine-1177 [11, 12]. EDHF, primarily intracellular K^+^, have been proposed as a novel vasoactive regulator of endothelium-dependent vasorelaxation. Intracellular K^+^ flows out through potassium channels of VECs and VSMCs, hyperpolarizes cellular membrane potential and relaxes vascular smooth muscle [13].

**Fig 1:**
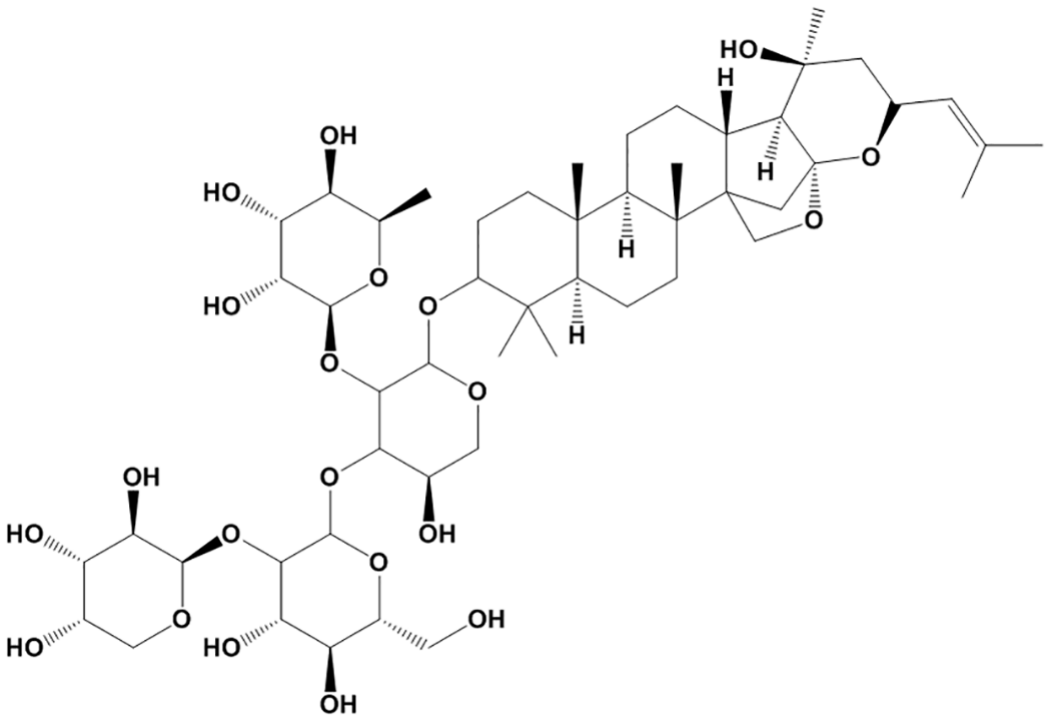
Structural formula of Jujuboside B.

Zizyphi Spinosi Semen (ZSS, Suanzaoren) is the dried mature seed of *Zizyphus jujube Mill of Rhamnaceae*. ZSS has been used to treat amnesia, neurasthenia, anxiety, and insomnia in traditional Chinese medicine [14]. Modern pharmacological studies revealed that ZSS possessed various cardioprotective effects, such as protecting cardiomyocytes from ischemic injury, lowering blood pressure, etc [15, 16]. ZSS contains various active components including saponins, triterpenoids, flavonoids, alkaloids and fatty acids [17, 18]. Jujuboside B is one of the most important saponins of ZSS and its content in ZSS is nearly 400 mg/kg (Fig 1) [19, 20]. Jujuboside B has been reported to suppress tumor formation and platelet aggregation. However, the vascular protective effect of Jujuboside B and the underlying mechanisms are poorly understood. In this study, we evaluated the effect of Jujuboside B on vascular tension and endothelial function and explored the potential mechanisms.

## Materials and Methods

### Animals

Male Sprague—Dawley rats (150–200 g) were purchased from Vital River Laboratory Animal Technology Co. (Beijing, China). Rats were housed under standard conditions for experimental animals (temperature 20 ~ 22°C, humidity 40% ~ 70%, 12/12 h light-dark cycle) and free to eat and drink. All animal protocols were approved by the ethic committees of Harbin Medical University and observed the rules of the National Institutes of Health.

### Measurement of vascular tension

Vascular reactivity of isolated thoracic aorta was measured by Wire myograph system (Danish Myo Technology, Denmark) as described [21]. Rats were anesthetized and thoracic aorta was removed and fastened with two syringe needles in cold Krebs buffer containing (in mM): NaCl 118, NaHCO_3_ 25, D-glucose 11, KCl 4.7, KH_2_PO_4_ 1.2, MgSO_4_ 1.17, and CaCl_2_ 2.5, pH 7.4. The thoracic aorta was cut into vascular rings (3–4 mm in length) after being cleared off adjacent connective tissues. Attention was paid not to damage the vascular endothelium. Vascular rings without endothelium were prepared by rubbing the vascular endothelium layer with cotton swabs slightly. Vascular rings were suspended on myograph and perfused in Krebs solution aerated with 95% O_2_ and 5% CO_2_ at 37°C. After stabilizing for 60 minutes, the rings were maintained at a resting tension of 2.0 g. Then the rings were constricted with 20 μM phenylephrine (PE, Shanghai Harvest Pharmaceutical Co. Ltd, Shanghai, China) and relaxed with 10 μM carbachol (Sigma-Aldrich, St Louis, USA) to test the integrity of endothelium. Segments relaxing more than 80% were considered as endothelium-intact, while those relaxing less than 20% were identified as endothelium-denuded. Then the rings were washed with Krebs buffer to basal tension. After a sustained contraction induced by PE, Jujuboside B (Tianjin Shilan Technology Ltd., Tianjin, China) was dissolved in distilled water and added cumulatively to the bath. In different experimental groups, tissues were incubated with 100 μM L-NAME, 10 μM indomethacin, 10 μM glibenclamide, 100 nM iberiotoxin, 10 nM 4-DAMP, 10 μM KN93, 200 μM EGTA and 10 μM SKF96365 for 15 min prior to being constricted with PE. L-NAME, indometacin, glibenclamide, iberiotoxin, 4-DAMP, EGTA, KN93 and SKF96365 were purchased from Sigma-Aldrich Corporation, St Louis, USA.

### Measurement of NO production

NO content was determined by Griess reaction assay in cultured human aortic endothelial cells (HAECs) (Sciencell Research Laboratories, San Diego, USA). Firstly, HAECs were incubated with Jujuboside B and the culture medium was collected at different time points. Then, NO content was determined by measuring total nitrites in culture medium with a nitrite detection kit (Beyotime Institute of Biotechnology, Shanghai, China). The concentration of nitrite was determined by visible spectrophotometry at 540 nm and calculated through a standard curve derived from NaNO_2_ (0–100 μM) [22].

### Measurement of eNOS activity

eNOS activity was measured by enzyme linked immunosorbent assay (IBL International, Hamburg, Germany). HAECs were grown on 6-well plate and pretreated with 100 μM L-NAME, 200 μM EGTA or 10 μM SKF96365 for 1 h. Then cells were incubated with Jujuboside B for 15 min and lyased with ice-cold cell lysis buffer containing protease inhibitor (Beyotime Institute of Biotechnology, Shanghai, China). The cell lysate was centrifuged at 13500 g for 15 min at 4°C and the supernatant was collected for eNOS determination [23].

### Western blotting Analysis

Protein levels were quantified by western blotting procedures. HAECs were pretreated with 200 μM EGTA or 10 μM SKF96365 before incubating with Jujuboside B 10 μM for 15 min. Then cells were lyased with ice-cold lysis buffer containing protease and phosphatise inhibitor and the lysate was centrifuged at 13500 g for 15 min at 4°C. The supernatant was collected and the protein concentration was determined by using bicinchoninic acid protein assay reagent kit (Beyotime Institute of Biotechnology, Shanghai, China). Samples were separated by electrophoresis on 8% SDS-PAGE gels and transferred onto polyvinylidene difluoride membranes. The residual protein bands were blocked with 5% milk and the membrane was incubated with rabbit p-eNOS (Serine-1177) antibody and rabbit eNOS antibody (Cell Signaling Technology, Inc, Boston, USA) dilution overnight. Then membrane was incubated with Horseradish peroxidase (HRP)-conjugated rabbit antibody (LI-COR Bioscience, Lincoln, USA) dilution for 1 h in darkness. Then protein levels were determined by Infrared Fluorescence Imaging Detector. To normalize the data, β-actin (Sangon Biotech, Shanghai, China) was used as an internal reference.

### Measurement of intracellular Ca^2+^ concentration

HAECs were seeded in the circular discs and pretreated with 200 μM EGTA or 10 μM SKF96365 for 1 h. Then, cells were incubated with Jujuboside B 10 μM and loaded with 5 μM Fluo-3/AM (Life Technologies, Carlsbad, USA) and 10 μM Pluronic F-127 for 45 min at 37°C in Tyrode’s solution containing (in mM): NaCl 140, KCl 5, CaCl_2_ 1, MgCl_2_ 1, glucose 10 and HEPES 5, pH 7.4. Intracellular Ca^2+^ concentration were evaluated by measuring the fluorescence intensity excited at 506 nm and emitted at 526 nm using laser scanning confocal microscope (Olympus FV-1000, Olympus Optical Co. Ltd, Japan). Dynamic changes of intracellular Ca^2+^ were also monitored to evaluate the regulatory effect of Jujuboside B on intracellular Ca^2+^ [24, 25]. HAECs were seeded in the circular discs and pretreated with 200 μM EGTA or 10 μM SKF96365 for 1h. Then cells were loaded with 5 μM Fluo-3/AM and 10 μM Pluronic F-127 for 45 min at 37°C in Tyrode’s solution. Scanning time was set as 1500 s and Jujuboside B was administrated into the discs at a fixed time point. In another experiment, thapsigargin was administrated into the discs to deplete the internal Ca^2+^ stores and then Jujuboside B was administrated into the discs. Fluorescence intensity was determined before and after administrating drugs.

### Data analysis

All data were shown as mean ± SEM. Relaxing ability of Jujuboside B was expressed as a percentage of decreasing vascular tension to the maximum contractile response induced by PE. Results were analyzed by using Student’s *t*-test and ANOVA analysis. A *P*-value of less than 0.05 was regarded to be significant.

## Results

### Jujuboside B reduced vascular tension by activating eNOS and increasing NO generation

**Fig 2:**
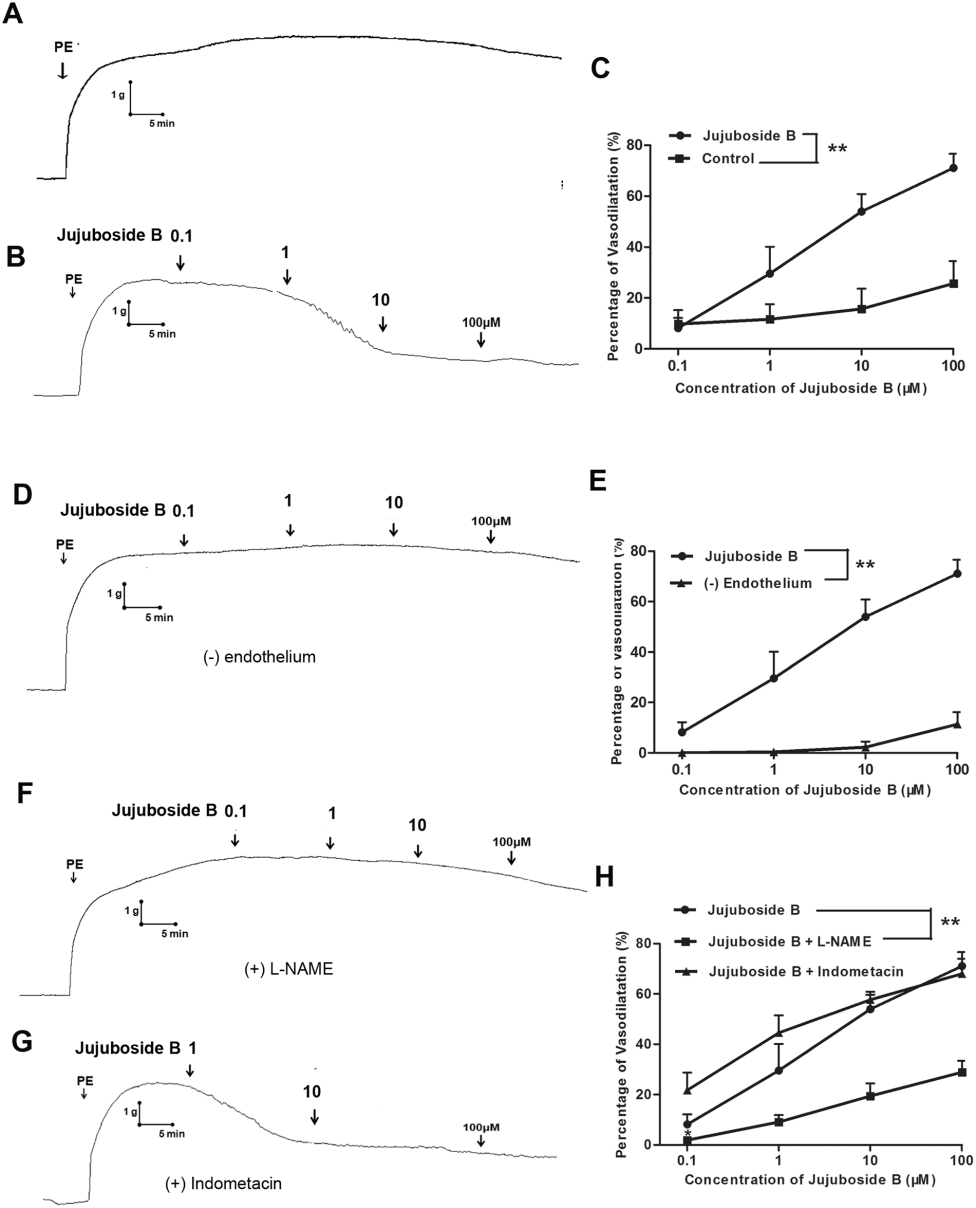
Jujuboside B reduced vascular tension of endothelium-intact vascular rings depending on eNOS activity. (A) Representative vascular tension trace showed that distilled water (served as Control) did not relax vascular rings preconditioned with PE. (B) Representative vascular tension trace showed that Jujuboside B relaxed vascular rings preconditioned with PE in a dose-dependent manner. (C) Dose-response curves showed that Jujuboside B significantly relaxed vascular rings preconditioned with PE in a dose-dependent manner. ** *P* < 0.01, n = 4. (D) Representative vascular tension trace showed that removing vascular endothelium significantly inhibited Jujuboside B-induced vasodilation in vascular rings preconditioned with PE. (E) Dose response curves showed that removing vascular endothelium significantly inhibited Jujuboside B-induced vasodilation. ** *P* <0.01, n = 4. (F) Representative vascular tension trace showed that L-NAME significantly inhibited Jujuboside B-induced vasodilation in endothelium-intact vascular rings preconditioned with PE. (G) Representative vascular tension trace showed that indometacin did not affect Jujuboside B-induced vasodilation in endothelium-intact vascular rings preconditioned with PE. (H) Dose response curves showed that L-NAME significantly inhibited Jujuboside B-induced vasodilation, while indometacin had no inhibitory effect. ** *P* <0.01, n = 4.

**Fig 3:**
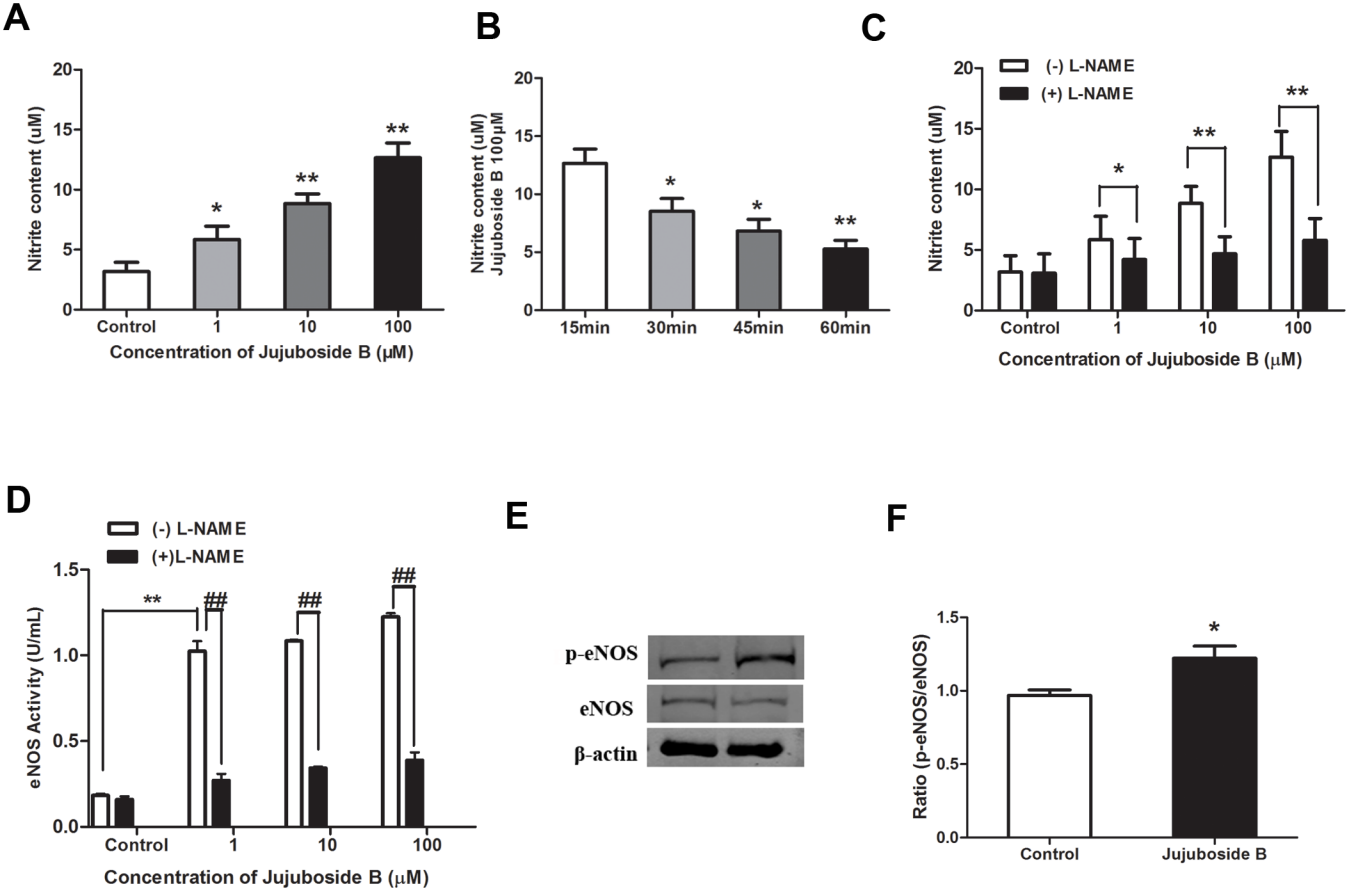
Jujuboside B increased NO generation and stimulated eNOS activation by phosphorylating eNOS at Serine-1177. (A)Jujuboside B increased NO generation in HAECs concentration-dependently. * *P* < 0.05, ** *P* < 0.01 compared with Control, n = 3. (B) Jujuboside B increased NO generation in HAECs negative time-dependently. * *P* < 0.05, ** *P* < 0.01 compared with 15 min group, n = 3. (C) L-NAME significantly inhibited Jujuboside B-induced NO generation. * *P* < 0.05, ** *P* < 0.01, n = 3. (D) L-NAME significantly inhibited Jujuboside B-induced eNOS activation. ** *P* < 0.01, ## *P* < 0.01, n = 4. (E) Western blot analysis of eNOS and p-eNOS at Serine-1177 in HAECs showed that Jujuboside B significantly increased p-eNOS Serine-1177 expression. (F) Densitometric analysis showed that Jujuboside B significantly increased p-eNOS Serine-1177 expression. * *P* < 0.05 compared with Control, n = 3.

In this study, we evaluated the effect of Jujuboside B on vascular tension and explored the underlying mechanisms. PE, a vasoconstrictor, constricted isolated rat thoracic aorta rings. Distilled water, served as Control, did not affect the tension of vascular rings preconditioned with PE (Fig 2A). Jujuboside B relaxed vascular rings preconditioned with PE in a dose-dependent manner compared with Control (Fig 2B and 2C). However, depriving vascular endothelium significantly inhibited Jujuboside B-induced vasodilation, suggesting that the vasodilatory effect of Jujuboside B was regulated by vascular endothelium (Fig 2D and 2E). Endothelium evokes vasorelaxation by releasing EDRFs. NO and PGI_2_ are the most important EDRFs that regulate vascular tension and associate with vasoactive factors to protect endothelial function [26]. In order to confirm the EDRFs participated in Jujuboside B-induced vasodilation, effects of NO and PGI_2_ on Jujuboside B-induced vasodilation were examined. Results showed that inhibiting NO generation with L-NAME significantly attenuated Jujuboside B-induced vasodilation, while inhibiting PGI_2_ generation with indometacin had no such effect (Fig 2F, 2G and 2H). Therefore, we speculated that Jujuboside B-induced vasodilation was related to eNOS activity and NO bioavailability in vascular endothelium. As expected, Jujuboside B increased NO generation in HAECs in a dose-dependent manner (Fig 3A). However, this effect was significantly decreased with the incubation time prolonging (Fig 3B). Besides, L-NAME significantly antagonized Jujuboside B-induced NO generation, confirming that Jujuboside B promoted NO generation and vasodilation depending on eNOS activity (Fig 3C). The effect of Jujuboside B on eNOS activity was determined since NO was generated exclusively by the catalysis of eNOS in VECs [27]. Results showed that Jujuboside B increased eNOS activity dose-dependently and this effect was significantly abolished by eNOS inhibitor L-NAME (Fig 3D). The protein level of p-eNOS Serine-1177 was detected since phosphorylation of eNOS at serine residue was supposed to be the most important manner to activate eNOS [27]. Likewise, Jujuboside B significantly increased p-eNOS Serine-1177 expression in HAECs (Fig 3E and 3F). These results showed that Jujuboside B-induced vasodilation was mainly attributed to Jujuboside B-induced eNOS activation and NO generation in VECs.

### Jujuboside B reduced vascular tension relating to extracellular Ca^2+^ and TRPC channels

**Fig 4:**
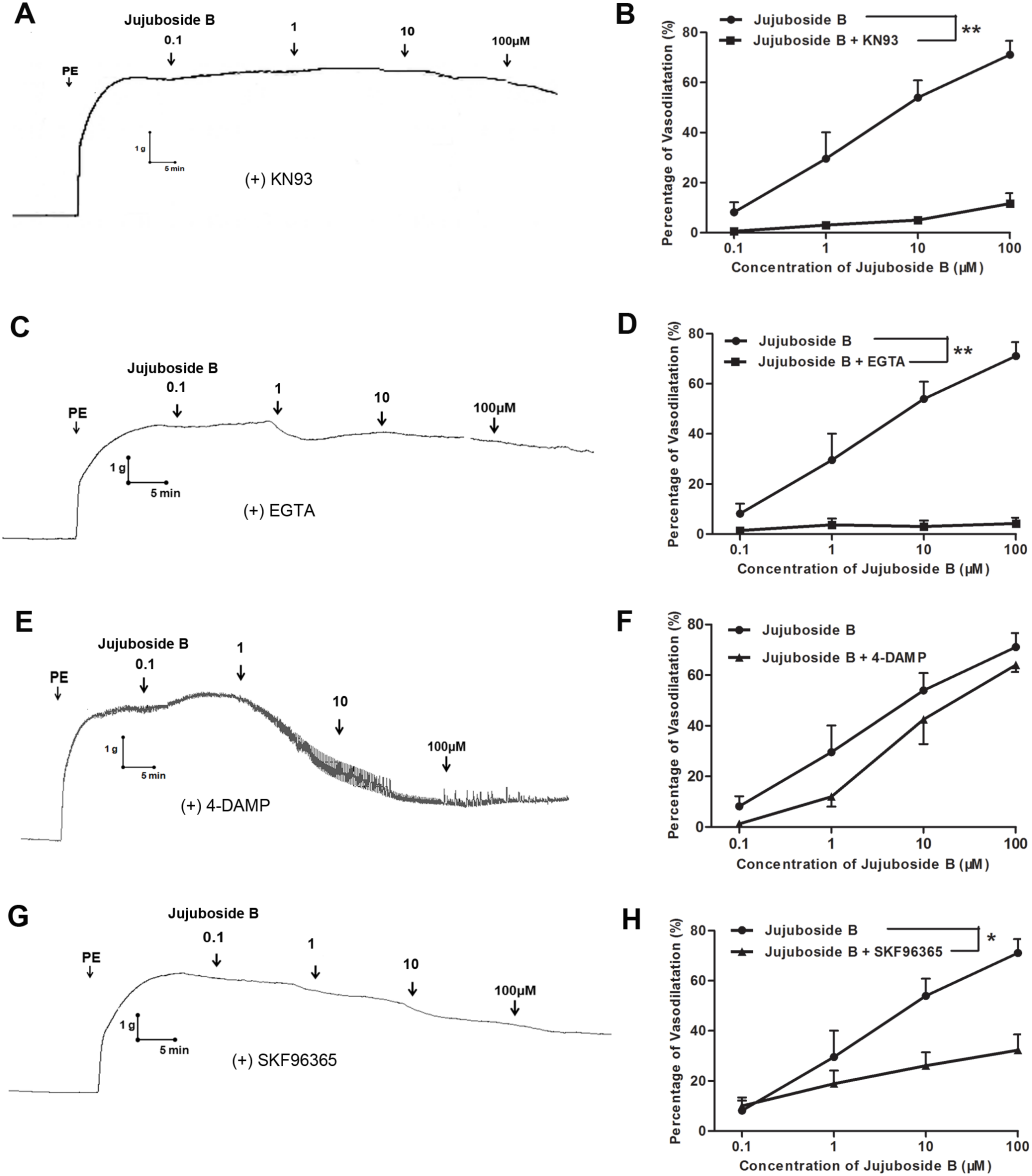
Jujuboside B reduced vascular tension depending on CaM kinase II activity, extracellular Ca^2+^ influx and TRPC channels. (A) Representative vascular tension trace showed that KN93 significantly inhibited Jujuboside B-induced vasodilation in endothelium-intact vascular rings preconditioned with PE. (B) Dose response curves showed that KN93 significantly inhibited Jujuboside B-induced vasodilation. ** *P* < 0.01, n = 4. (C) Representative vascular tension trace showed that EGTA significantly inhibited Jujuboside B-induced vasodilation in endothelium-intact vascular rings preconditioned with PE. (D) Dose response curves showed that EGTA significantly inhibited Jujuboside B-induced vasodilation. ** *P* <0.01, n = 4. (E) Representative vascular tension trace showed that 4-DAMP did not inhibit Jujuboside B-induced vasodilation in endothelium-intact vascular rings preconditioned with PE. (F) Dose response curves showed that 4-DAMP did not inhibit Jujuboside B-induced vasodilation, n = 4. (G) Representative vascular tension trace showed that SKF96365 significantly inhibited Jujuboside B-induced vasodilation in endothelium-intact vascular rings preconditioned with PE. (H) Dose response curves showed that SKF96365 significantly inhibited Jujuboside B-induced vasodilation. * *P* < 0.05, n = 4.

**Fig 5:**
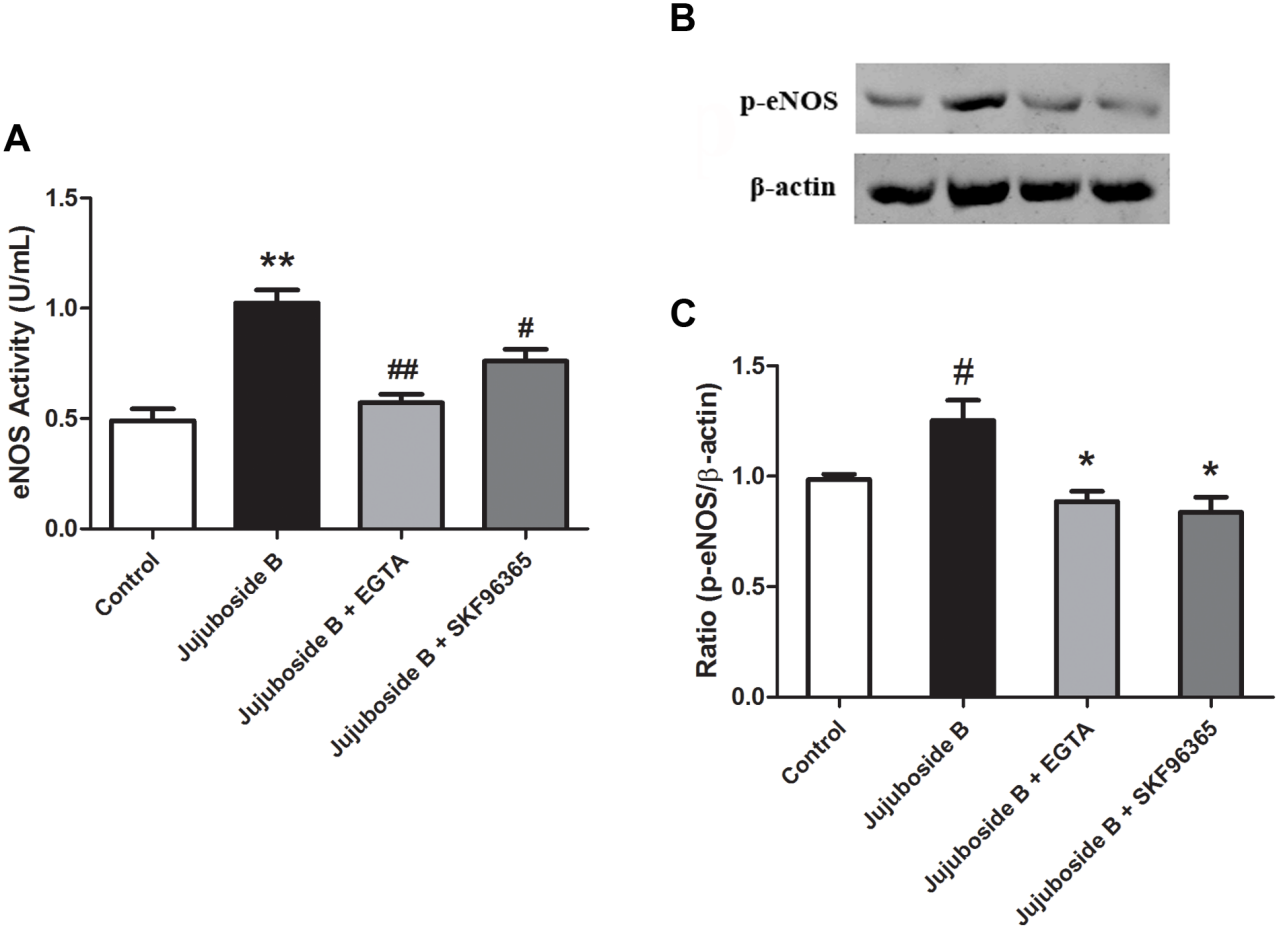
Jujuboside B activated eNOS depending on extracellular Ca^2+^ and TRPC channels. (A)EGTA and SKF96365 significantly inhibited Jujuboside B-induced eNOS activation. ** *P* < 0.01 compared with Control, # *P* < 0.05, ## *P* <0.01 compared with Jujuboside B, n = 4. (B) Western blot analysis showed that EGTA and SKF96365 significantly inhibited Jujuboside B-induced eNOS phosphorylation at Serine-1177. (C) Densitometric analysis showed that EGTA and SKF96365 significantly inhibited Jujuboside B-induced eNOS phosphorylation at Serine-1177. # *P* < 0.05 compared with Control, * *P* < 0.05 compared with Jujuboside B, n = 3.

Since eNOS is classified as a strict Ca^2+^/CaM-dependent enzyme, it can be activated by Ca^2+^/CaM complex and phosphorylated by CaM kinase II after intracellular Ca^2+^ increases [28, 29]. Our results showed that inhibiting CaM kinase II with KN93 significantly inhibited Jujuboside B-induced vasodilation, suggesting that Jujuboside B reduced vascular tension depending on CaM kinase II activity which was regulated by intracellular Ca^2+^ (Fig 4A and 4B). Intracellular Ca^2+^ derived mainly from extracellular Ca^2+^ influxing through transmembrane calcium channels and Ca^2+^ releasing from internal Ca^2+^ stores through agitated membrane receptors, especially endothelial M_3_ muscarine receptor [10]. Our results showed that chelating extracellular Ca^2+^ with EGTA significantly inhibited Jujuboside B-induced vasodilation (Fig 4C and 4D), while antagonizing M_3_ muscarine receptor with 4-DAMP had no such effect, indicating that Jujuboside B reduced vascular tension mainly depending on extracellular Ca^2+^ influx(Fig 4E and 4F). However, VECs barely express functional voltage-gated calcium channels [30]. In VECs, transient receptor potential cation (TRPC) channels are major ion channels accounting for agonist-induced extracellular Ca^2+^ influx and regulating endothelial function [31]. Our results showed that blocking extracellular Ca^2+^ influx through TRPC channels with SKF96365 significantly inhibited Jujuboside B-induced vasodilation(Fig 4G and 4H). Moreover, EGTA and SKF96365 inhibited Jujuboside B-induced eNOS activation and phosphorylation, indicating that Jujuboside B-induced vasodilation and eNOS activation mainly resulted from extracellular Ca^2+^ influx and TRPC channels (Fig 5).

### Jujuboside B increased extracellular Ca^2+^ influx through TRPC channels

**Fig 6:**
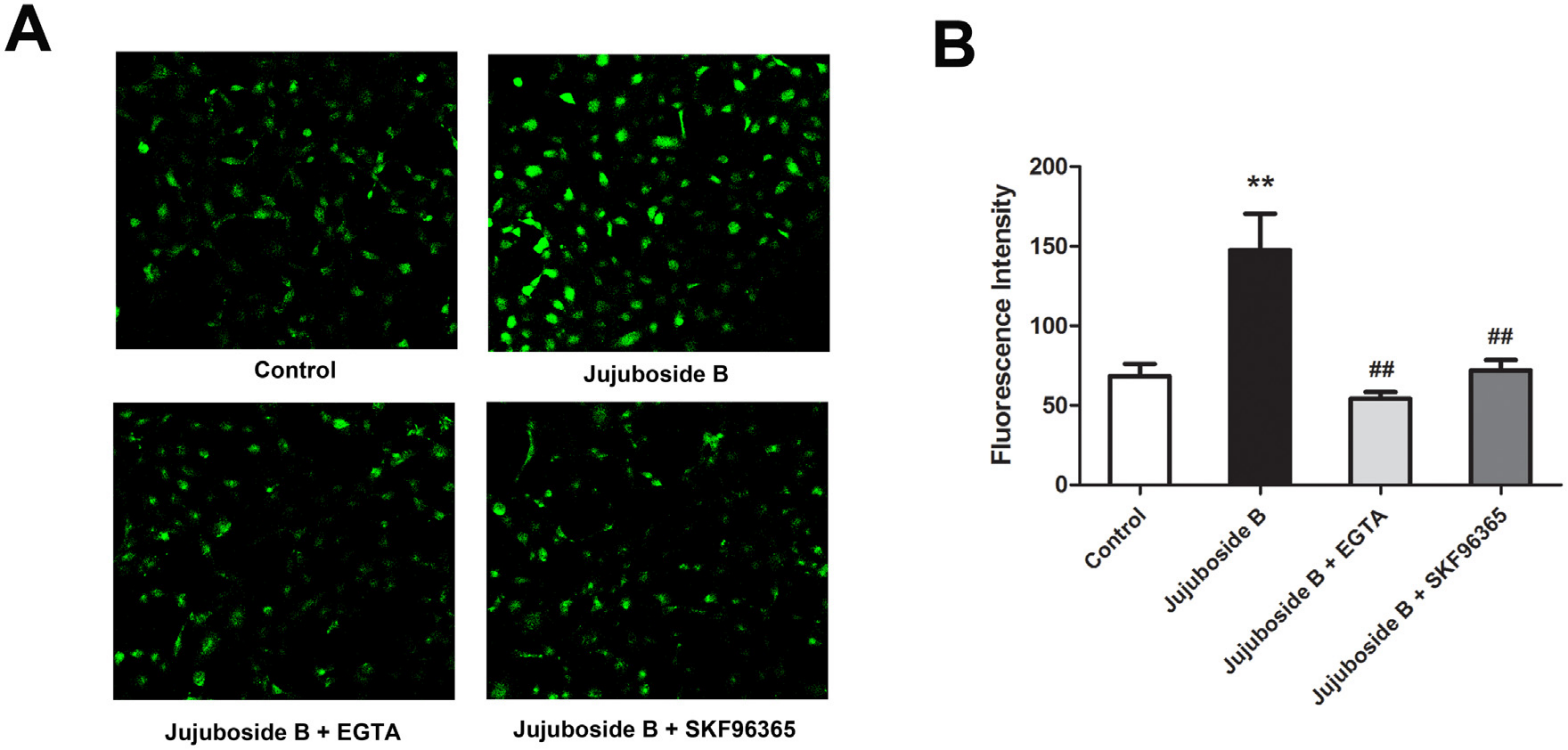
Jujuboside B increased intracellular Ca^2+^ concentration in HAECs. (A) Representative confocal image showed that Jujuboside B increased intracellular Ca^2+^ concentration in HAECs and this effect was reversed by EGTA and SKF96365. (B) Fluorescence density analysis showed that Jujuboside B increased intracellular Ca^2+^ in HAECs and this effect was reversed by EGTA and SKF96365, ** *P* < 0.01 compared with Control, ## *P* < 0.01 compared with Jujuboside B, n = 4.

**Fig 7:**
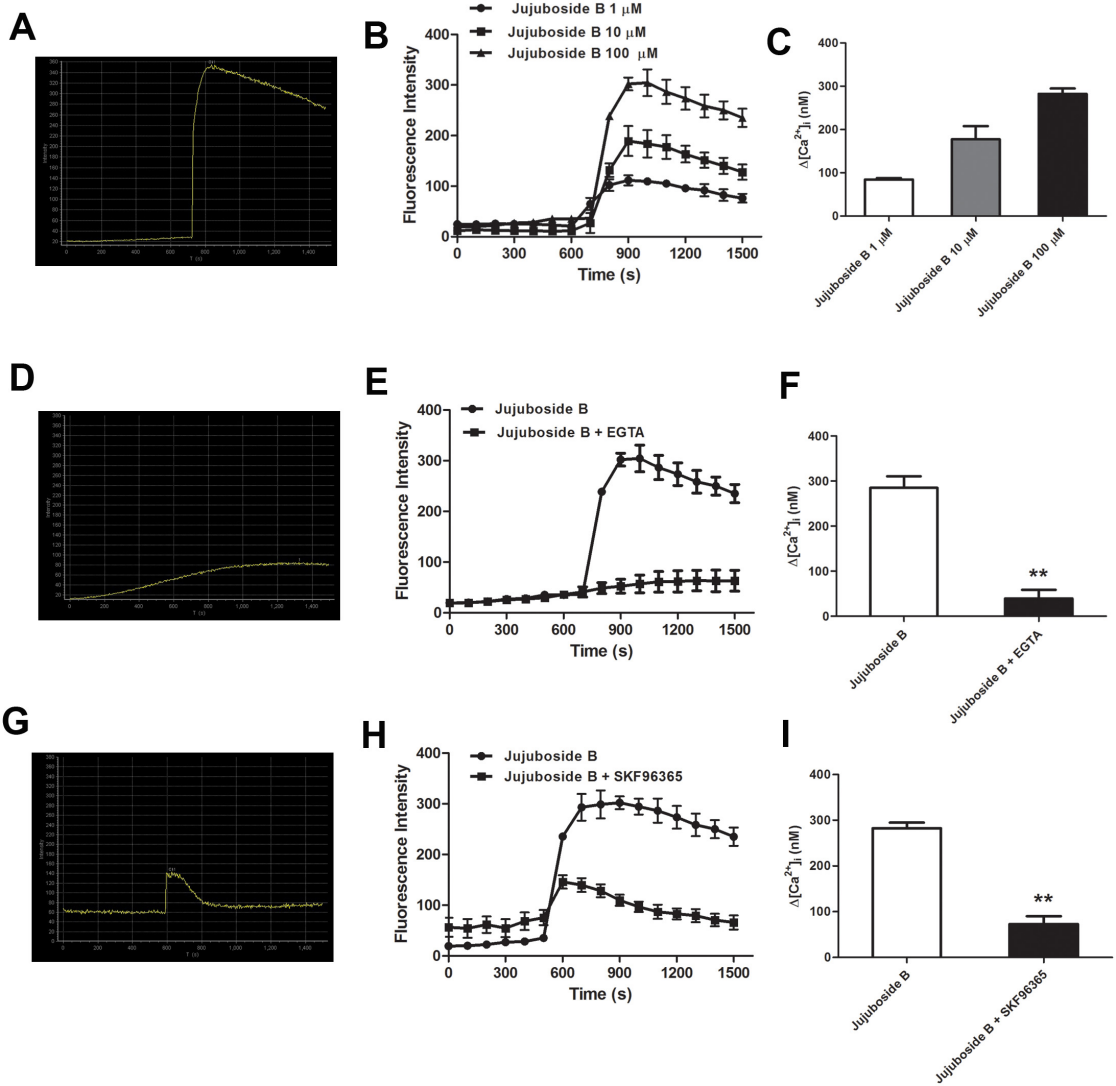
Jujuboside B increased intracellular Ca^2+^ by inducing extracellular Ca^2+^ to inflow through TRPC channels. (A) Representative confocal image showed that Jujuboside B induced a rapid intracellular Ca^2+^ elevation in HAECs instantaneously. (B) Fluorescence intensity curves showed that Jujuboside B increased intracellular Ca^2+^ concentration in a dose-dependent manner, n = 4. (C) Analysis of intracellular Ca^2+^ concentration showed that Jujuboside B increased intracellular Ca^2+^ concentration in a dose-dependent manner, n = 4. (D) Representative confocal image showed that EGTA inhibited Jujuboside B-induced intracellular Ca^2+^ increase. (E) Fluorescence intensity curves showed that EGTA significantly inhibited Jujuboside B-induced intracellular Ca^2+^ increase, n = 4. (F) Analysis of intracellular Ca^2+^ concentration showed that EGTA significantly inhibited Jujuboside B-induced intracellular Ca^2+^ increase, ** *P* < 0.01, n = 4. (G) Representative confocal image showed that SKF96365 inhibited Jujuboside B-induced intracellular Ca^2+^ increase. (H) Fluorescence intensity curves showed that SKF96365 significantly inhibited Jujuboside B-induced intracellular Ca^2+^ increase, n = 4. (I) Analysis of intracellular Ca^2+^ concentration showed that SKF96365 significantly inhibited Jujuboside B-induced intracellular Ca^2+^ increase, ** *P* < 0.01, n = 4.

**Fig 8:**
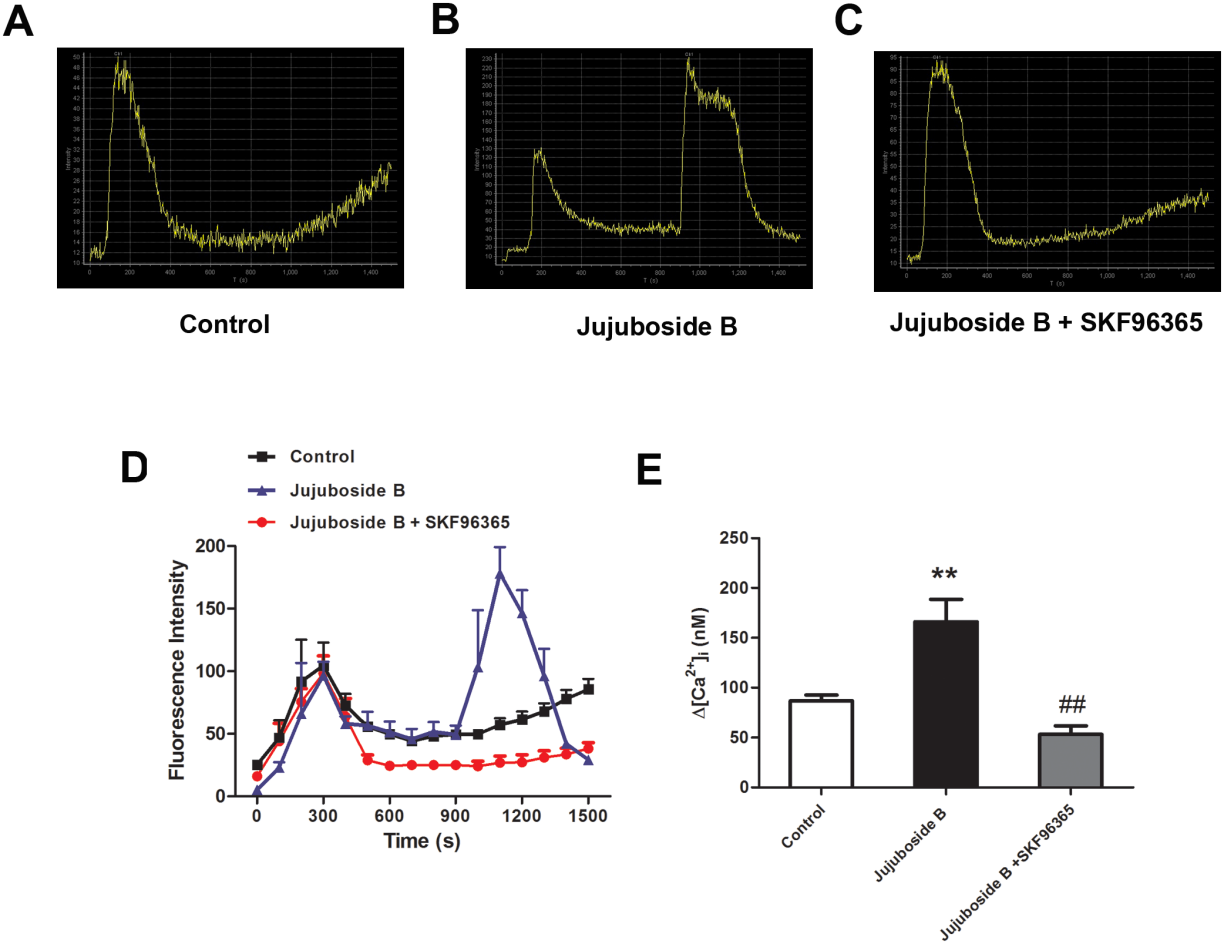
Jujuboside B increased Ca^2+^ influx through TRPC channels. (A) Representative confocal image showed that depleting intracellular Ca^2+^ store by thapsigargin initiated a slow and continuous Ca^2+^ influx. (B) Representative confocal image showed that after depleting intracellular Ca^2+^ store by thapsigargin, instantaneous administration of Jujuboside B induced a rapid and intense Ca^2+^ influx. (C) Representative confocal image showed that after depleting intracellular Ca^2+^ store by thapsigargin, SKF96365 inhibited Jujuboside B-induced Ca^2+^ influx. (D) Fluorescence intensity curves showed that SKF96365 significantly inhibited Jujuboside B-induced intracellular Ca^2+^ elevation, n = 4. (E) Analysis of intracellular Ca^2+^ concentration showed that SKF96365 significantly inhibited Jujuboside B-induced intracellular Ca^2+^ elevation, ** *P* < 0.01 compared with Control, ## *P* < 0.01 compared with Jujuboside B, n = 4.

Classified as Ca^2+^/CaM dependent enzyme, eNOS can be stimulated by increasing intracellular Ca^2+^ in VECs. Ca^2+^ activates CaM, which binds to the CaM-binding domain of eNOS, activates CaM kinase II and phosphorylates eNOS at Serine-1177 [32]. In this study, we proved that Jujuboside B induced eNOS activation and vasodilation in a Ca^2+^-dependent manner. Then, we explored the regulatory effect of Jujuboside B on intracellular Ca^2+^ concentration in VECs. Results showed that Jujuboside B significantly increased intracellular Ca^2+^ concentration in HAECs. EGTA abolished Jujuboside B-induced intracellular Ca^2+^ elevation, suggesting that Jujuboside B increased intracellular Ca^2+^ concentration by inducing extracellular Ca^2+^ influx. Moreover, SKF96365 significantly inhibited Jujuboside B-induced intracellular Ca^2+^ elevation, suggesting that Jujuboside B induced extracelular Ca^2+^ influx probably through TRPC channels (Fig 6). Dynamic determination of intracellular Ca^2+^ also showed that Jujuboside B increased intracellular Ca^2+^ concentration instantaneously in a dose-dependent manner. EGTA and SKF96365 significantly inhibited Jujuboside B-stimulated intracellular Ca^2+^ increase, further confirming our conclusion that Jujuboside B induced intracellular Ca^2+^ elevation depending on extracellular Ca^2+^ influx through TRPC channels (Fig 7). TRPC channels are classified as receptor or store-operated calcium channels which can be stimulated by depleting intracellular Ca^2+^ stores [33]. Our results showed that after depleting endoplasmic reticulum Ca^2+^ stores with thapsigargin, instantaneous administration of Jujuboside B induced a rapid intracellular Ca^2+^ elevation immediately compared with Control, while SKF96365 significantly inhibited Jujuboside B-induced intracellular Ca^2+^ elevation (Fig 8). The results suggested that Jujuboside B-stimulated intracellular Ca^2+^ elevation was induced by extracellular Ca^2+^ inflowing through TRPC channels.

### Jujuboside B reduced vascular tension involving endothelium-dependent hyperpolarization

**Fig 9:**
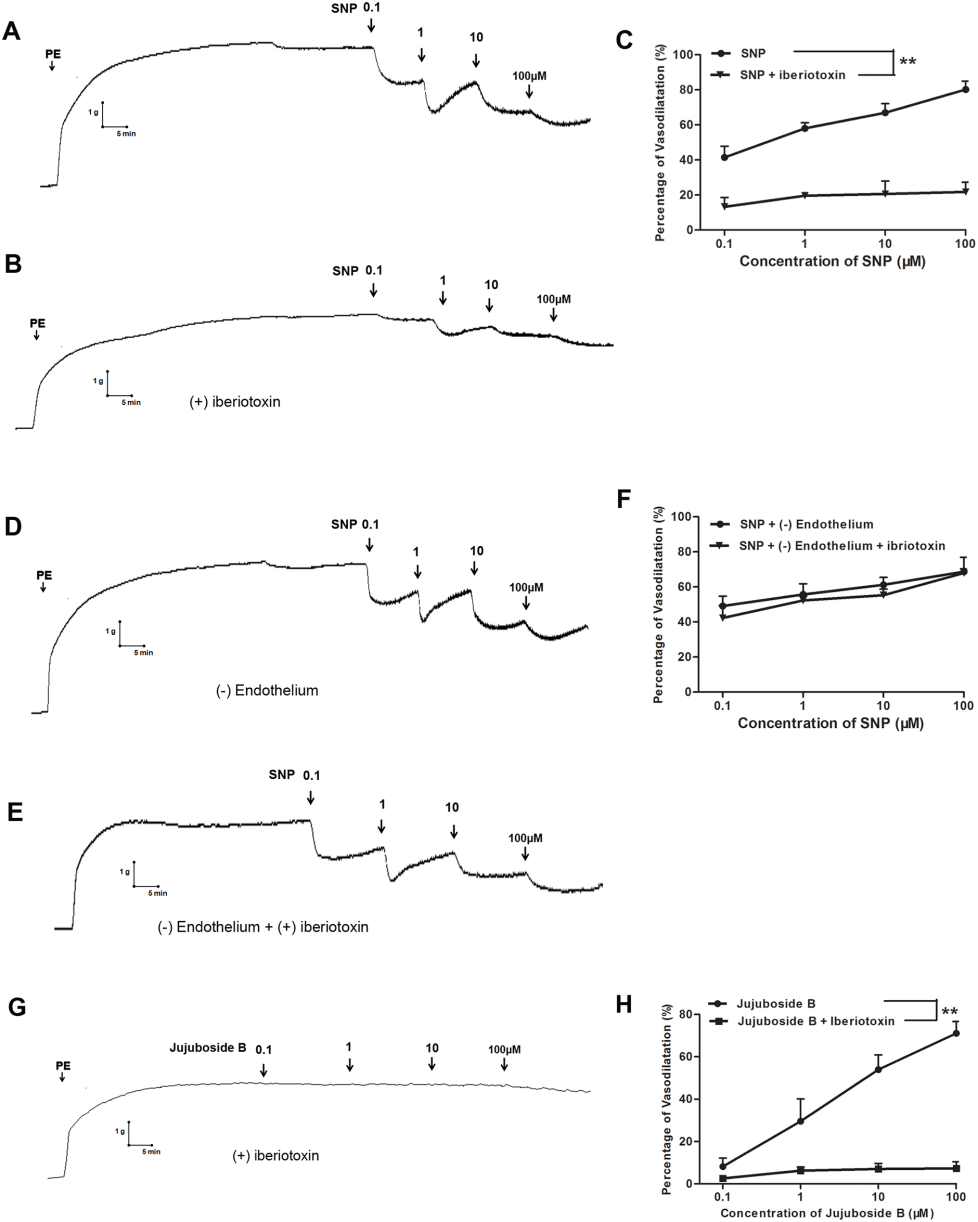
Jujuboside B reduced vascular tension involving endothelium dependent hyperpolarization through endothelial BK_Ca_ channels. (A)Representative vascular tension trace showed that SNP reduced vascular tension of endothelium-intact vascular rings preconditioned with PE in a dose-dependent manner. (B) Representative vascular tension trace showed that iberiotoxin significantly inhibited SNP-induced vasodilation in endothelium-intact vascular rings. (C) Dose response curves showed that iberiotoxin significantly inhibited SNP-induced vasodilation, ** *P* < 0.01, n = 3. (D) Representative vascular tension trace showed that SNP significantly reduced vascular tension of endothelium-denuded vascular rings preconditioned with PE in a dose-dependent manner. (E) Representative vascular tension trace showed that iberiotoxin did not inhibited SNP-induced vasodilation in endothelium-denuded vascular rings. (F) Dose response curves showed that iberiotoxin did not affect SNP-induced vasodilation in endothelium-denuded vascular rings, n = 3. (G) Representative vascular tension trace showed that iberiotoxin significantly inhibited Jujuboside B-induced vasodilation in endothelium-intact vascular rings preconditioned with PE. (H) Dose response curves showed that iberiotoxin significantly inhibited Jujuboside B-induced vasodilation, ** *P* < 0.01, n = 4.

**Fig 10:**
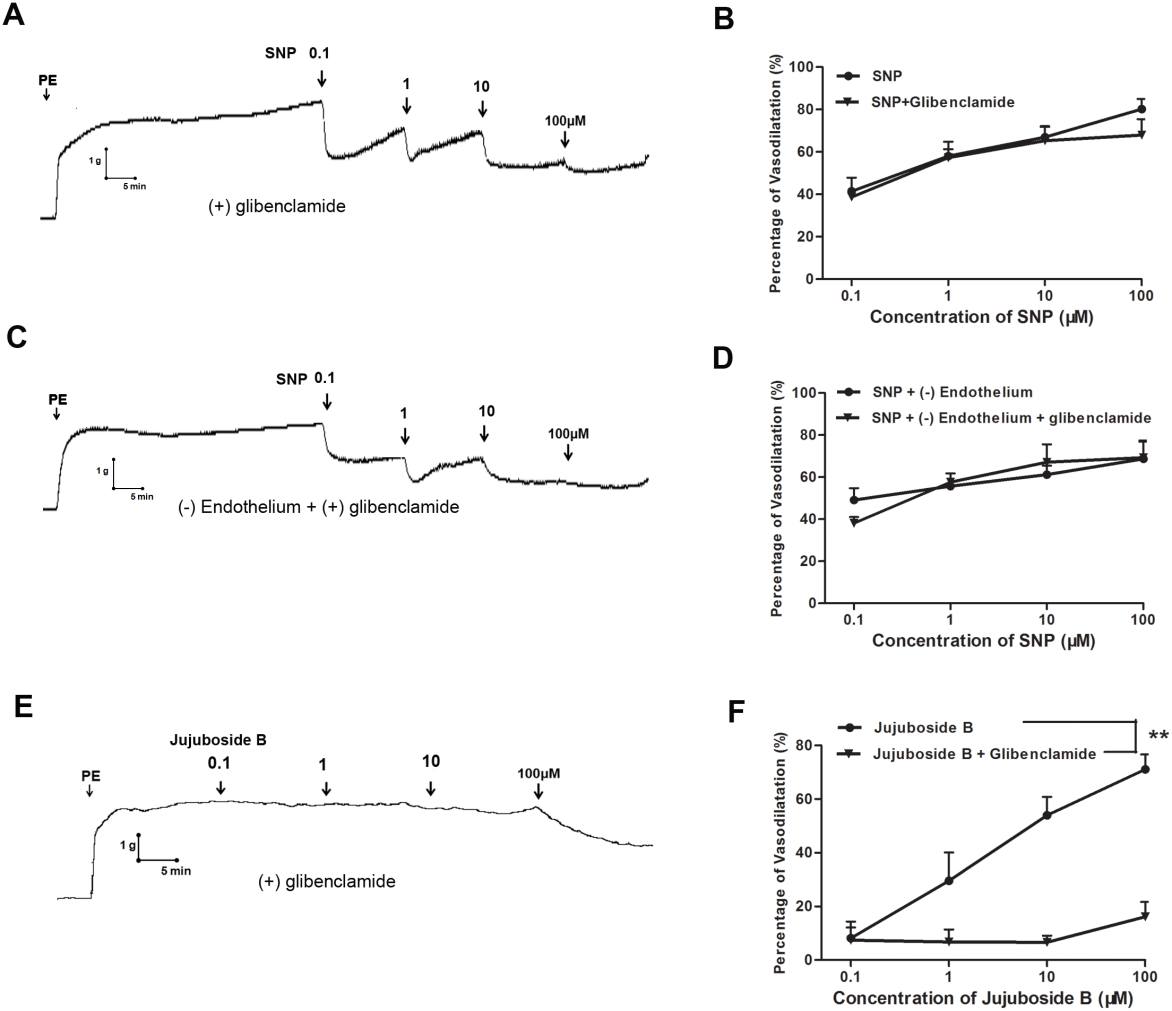
Jujuboside B reduced vascular tension involving endothelium dependent hyperpolarization through endothelial K_ATP_ channels. (A)Representative vascular tension trace showed that glibenclamide did not inhibit SNP-induced vasodilation in endothelium-intact vascular rings preconditioned with PE. (B) Dose response curves showed that glibenclamide did not affect SNP-induced vasodilation, n = 3. (C) Representative vascular tension trace showed that glibenclamide did not inhibit SNP-induced vasodilation in endothelium-denuded vascular rings preconditioned with PE. (D) Dose response curves showed that glibenclamide did not affect SNP-induced vasodilation in endothelium-denuded vascular rings, n = 3. (E) Representative vascular tension trace showed that glibenclamide significantly inhibited Jujuboside B-induced vasodilation in endothelium-intact vascular rings preconditioned with PE. (F) Dose response curves showed that glibenclamide significantly inhibited Jujuboside B-induced vasodilation, ** *P* < 0.01, n = 4.

Potassium channels expressed on vasculature participate in regulating vascular function through modulating membrane potential and induce endothelium-dependent hyperpolarization by regulating the motion of K^+^ [13]. In this study, sodium nitroprusside (SNP) was adopted as a positive control drug to explore the endothelium-dependent hyperpolarization involved in Jujuboside B-induced vasodilation. SNP is a well-known vasodilator which is widely used in acute cardiovascular incident, such as hypertensive crisis and acute left ventricular failure. SNP is a water-soluble sodium salt which functions as a prodrug and reacts with sulfhydryl groupson to release NO in VSMCs independent of vascular endothelium [34]. Our results showed that SNP reduced the tension of vascular rings with and without endothelium in a dose-dependent manner. Its intense vasodilatory effect was different from Jujuboside B (Fig 9A and 9D). Besides, iberiotoxin significantly inhibited SNP-induced vasodilation in endothelium-intact vascular rings, suggesting that SNP reduced vascular tension relating to opening BK_Ca_ channels (Fig 9B and 9C). However, iberiotoxin did not inhibit SNP-induced vasodilation in endothelium-denuded vascular rings, suggesting that SNP reduced vascular tension relating to opening endothelial BK_Ca_ channels (Fig 9E and 9F). Compared with SNP, iberiotoxin significantly inhibited Jujuboside B-induced vasodilation in endothelium-intact vascular rings, suggesting that Jujuboside B reduced vascular tension relating to opening endothelial BK_Ca_ channels (Fig 9G and 9H). In another experiment, glibenclamide did not inhibit SNP-induced vasodilation either in endothelium-intact or in endothelium-denuded vascular rings, suggesting that SNP reduced vascular tension independent of opening K_ATP_ channels. However, glibenclamide significantly attenuated Jujuboside B-induced vasodilation in endothelium-intact vascular rings, suggesting that Jujuboside B reduced vascular tension relating to opening endothelial K_ATP_ channels (Fig 10). These results indicated that the vasodilatory effect of Jujuboside B was related to endothelium-dependent hyperpolarization induced by opening endothelial BK_Ca_ and K_ATP_ channels.

## Discussion and Conclusions

ZSS is a one of the most commonly used sedative and hypnotic Chinese herbs with cardiovascular protective effect [15]. The present study provided experimental evidences of the vascular protective effect of Jujuboside B on reducing vascular tension, protecting endothelial function and the underlying mechanisms. The protective effects involved that Jujuboside B induced Ca^2+^ influx through TRPC channels, increased eNOS activity and NO release in VECs and thus relaxed blood vessels. Besides, Jujuboside B reduced vascular tension probably relating to endothelium dependent hyperpolarization through endothelial BK_Ca_ channels and K_ATP_ channels. Therefore, Jujuboside B is a potential compound to be applied in the research and development of drugs to treat endothelial dysfunction and vascular diseases.

Vascular endothelium works as a protective barrier between vascular tissues and circulating blood. It functions to maintain vascular homeostasis by releasing bioactive factors in response to hemodynamic changes and neurohumoral signals [26]. Jujuboside B reduced vascular tension of rat isolated thoracic aorta with intact endothelium concentration-dependently, indicating that the vasodilatory effect of Jujuboside B involved endothelium-dependent mechanisms. Meanwhile, various inhibitors were adopted to explore the EDRFs involved in Jujuboside B-induced vasodilation. L-NAME, an eNOS inhibitor, almost totally inhibited Jujuboside B-induced vasodilation, which was similar to the endothelium-denuded preparations. COX inhibitor indometacin had no inhibitory effect. These findings suggested that Jujuboside B-induced endothelium-dependent vasodilation depended on endothelial NO in endothelium-intact arteries, while endothelial PGI_2_ might be not involved. NO is the most recognized EDRFs which plays an important role in many physiological processes especially in regulating vascular tone and preventing endothelial dysfunction [35]. Recent studies also showed that when arteries were exposed to NO, endothelium-dependent contractions were inhibited [36]. Conversely, dyspoiesis of NO triggered many serious problems, including shock, infarction and vascular inflammation, etc [37]. In this study, we proved that Jujuboside B promoted NO release concentration-dependently in HAECs. However, Jujuboside B-induced NO release gradually decreased with incubation time prolonged, indicating that Jujuboside B-induced NO release was transient and moderate. Many clinical effective vasodilators induce vasodilation by releasing NO directly, such as nitroglycerin and SNP. However, continuous medication of such medicines brings about many clinical problems due to desensitizing the target enzyme guanylyl cyclase, activating rennin-angiotensin system and increasing catecholamine and vasopressin levels, which predispose blood vessels to attenuated vasodilator potency, crosstolerance to other endothelium-dependent vasodilators and increased oxidative stress indirectly [38]. Jujuboside B induced vasodilation by promoting NO release in VECs rather than directly transforming into NO in VSMCs. Therefore, the vasodilatory effect of Jujuboside B was much more moderate and was not prone to be harmful to vasculature and vascular function after long-term application. Moreover, the release of NO played a critical role in maintaining the normal function and redox homeostasis of vascular endothelium, which helped to prevent endothelial dysfunction and strengthen the capability of resisting stress of vascular tissues.

Since endothelial NO is generated exclusively through eNOS in VECs, endothelium-dependent vasodilation is closely linked to eNOS activity [39]. It is reported that serine phosphorylation of eNOS is essential for activating eNOS [28]. Our results showed that Jujuboside B activated eNOS and phosphorylated eNOS at Serine-1177 in HAECs, indicating that Jujuboside B increased NO release probably by phosphorylating eNOS and thus activating it. Classified as Ca^2+^/CaM enzyme, the activation of eNOS depends on the presence of Ca^2+^, CaM and CaM kinase II which are regulated by the changes of intracellular Ca^2+^ concentration [29, 30]. Therefore, eNOS can be activated and phophorylated by substances that increase intracellular Ca^2+^ levels. In addition to Ca^2+^ and CaM kinase II, some other protein kinases may regulate eNOS activity. Adenosine monophosphate protein kinase (AMPK) has been shown to phosphorylate and activate eNOS at Serine-1177 in cultured VECs and induce NO generation subsequently in response to hypoxia, metformin, adiponectin and shear stress [40]. AICAR, an AMPK activatior, has been reported to relax blood vessels by promoting NO generation and enhancing endothelium-dependent vasodilation [41]. Activation of PKA by increasing intracellular cyclic adenosine monophosphate (cAMP) also leads to phosphorylation of eNOS at Serine-1177 [42]. Research showed that forskolin, a cAMP activator, induced vasodilation by enhancing eNOS activity and NO release through activating PKA signal transduction [43]. Relevant research also reported that the activation of Serine/Threonine kinase phosphatidyl inositol 3-kinase (PI_3_K) by many vasoactive factors such as vascular endothelial growth factor (VEGF) and insulin-like growth factor-1 (IGF-1) stimulated protein kinase B (PKB/Akt) and phosphorylated eNOS at Serine-1177 [44, 45]. Our results showed that inhibiting PKA with H89, AMPK with P5499 and PI_3_K with LY294002 had little reverse effect on Jujuboside B-induced vasodilation (S1 Fig), while inhibiting CaM kinase II significantly attenuated Jujuboside B-induced vasodilation, suggesting that Jujuboside B-induced vasodilation was probably Ca^2+^/CaM-dependent. Ca^2+^/CaM complex can be activated by increased intracellular Ca^2+^ in VECs [46]. The sustained extracellular Ca^2+^ inflowing into the cell contributes to increasing intracellular Ca^2+^, which is necessary for the synthesis and release of NO in VECs [30]. Besides, activation of M_3_ muscarine receptor on endothelium increases intracellular Ca^2+^ by stimulating endoplasmic reticulum to release Ca^2+^ and thus induces NO generation and vasodilation [47]. Antagonizing muscarine receptor did not affect Jujuboside B-induced vasodilation, while chelating extracellular Ca^2+^ with EGTA significantly inhibited Jujuboside B-induced vasodilation. Meanwhile, EGTA significantly impaired Jujuboside B-induced eNOS activation and phosphorylation in HAECs, suggesting that Jujuboside B-induced NO production and vasodilation depending on extracellular Ca^2+^. To further verify this effect of Jujuboside B, intracellular Ca^2+^ concentration was measured. Results showed that Jujuboside B significantly increased intracellular Ca^2+^ in HAECs. Removing extracellular Ca^2+^ with EGTA significantly inhibited Jujuboside B-induced intracellular Ca^2+^ elevation, confirming that Jujuboside B-induced vasodilation and eNOS activation were due to the increase of intracellular Ca^2+^ through extracellular Ca^2+^ inflowing.

Numerous studies demonstrated that endothelial TRPC channels modulated intracellular Ca^2+^ levels in response to vasoactive agents. Malfunction of TRPC channels impaired Ca^2+^ influx, reduced NO bioavailability and suppressed vascular relaxation [31]. VECs express multiple transcripts of TRPC channels, which are involved in the agonist-activated Ca^2+^ entry and vasodilation. There are almost no functional voltage-dependent Ca^2+^ channels existing on VECs [30]. Previous studies showed that Ca^2+^ inflowing through TRPC channels was required for phosphorylating eNOS and producing NO in VECs because the stimulatory effect was inhibited by removing extracellular Ca^2+^ and a selective TRPC blocker [48]. In this study, blocking TRPC channels with SKF96365 significantly decreased Jujuboside B-induced vasodilation, eNOS activation and intracellular Ca^2+^ elevation in HAECs, suggesting that Jujuboside B-induced vasodilation and eNOS activation were related to inducing Ca^2+^ to inflow into cells through TRPC channels. As TRPC channels are classified as store or receptor-operated calcium channels, they can be stimulated by depleting internal Ca^2+^ stores. That is to say, TRPC channels can be activated by the endoplasmic reticulum Ca^2+^-ATPase inhibitor thapsigargin [30, 31]. However, in the absence of specific agonist of TRPC channels, the activation of TRPC channels by depleting intracellular Ca^2+^ stores induced a small and continuous Ca^2+^ influx spontaneously. While agonist-induced Ca^2+^ influx through TRPC channels was recognized as a rapid Ca^2+^ influx [49]. Our results showed that depleting Ca^2+^ stores with thapsigargin initiated a weak and continuous Ca^2+^ influx, while Jujuboside B induced a rapid extracellular Ca^2+^ influx. Meanwhile, blocking TRPC channels with SKF96365 significantly inhibited Jujuboside B-induced extracellular Ca^2+^ influx, confirming that Jujuboside B-induced intracellular Ca^2+^ elevation was attributed to inducing extracellular Ca^2+^ influx through activating TRPC channels. While, the specific mechanisms involved in this process need to be researched further.

Opening of potassium channels on vasculature with resultant hyperpolarization is another fundamental mechanism of vasodilation. Vasculature expresses various potassium channels to reduce vascular tension by inducing membrane hyperpolarization, including voltage-dependent potassium channel (K_V_), calcium-activated potassium channel (K_Ca_) and ATP-sensitive potassium channel (K_ATP_). According to the different channel conductance, K_Ca_ channels are classified into big conductance K_Ca_ channel (BK_Ca_), intermediate conductance K_Ca_ channel (IK_Ca_) and small conductance K_Ca_ channel (SK_Ca_) [50]. K_V_ channel contributes to stabilizing vascular tone and participates in cAMP-mediated vasodilation. Endothelial K_Ca_ channel can be stimulated by intracellular Ca^2+^ to hyperpolarize endothelial membrane. This effect can be conducted through myoendothelial gap junctions to hyperpolarize and relax adjacent vascular smooth muscle [51, 52]. Endothelial BK_Ca_ channel is a major target for vasoactive substances to trigger extracellular Ca^2+^ influx and induce memebrane hyperpolarizaiton. BK_Ca_ channel on VSMCs also participated in the cGMP-mediated vasodilaion induced by exogenous nitrovasodilators and EDRFs. BK_Ca_ channel can be blocked by iberiotoxin high-selectively [53, 54]. K_ATP_ channel play an important role in metabolic hyperpolarization and can be activated by decreased intracellular ATP level or cellular phosphorylation reaction. K_ATP_ channel can be blocked by glibenclamide [55, 56]. Besides, BK_Ca_ and K_ATP_ channel also participate in stabilizing membrane potential and regulating cellular membrane excitability according to intracellular Ca^2+^ and ATP concentration [13, 57]. Since our above results showed that Jujuboside B increased endothelial intracellular Ca^2+^ and phosphorylated eNOS, the involvement of BK_Ca_ and K_ATP_ channel, which were activated by intracellular Ca^2+^ elevation and phosphorylation, in Jujuboside B-induced vasodilation was examined. At the same time, SNP was adopted as a positive control drug to discuss the mechanisms of Jujuboside B-induced hyperpolarization. SNP is an endothelium-independent vasodilator which reduces vascular tension by relaxing vascular smooth muscle directly. Results showed that iberiotoxin significantly attenuated SNP-induced vasodilation in endothelium-intact vascular rings but not endothelium-denuded vascular rings, indicating that SNP-induced vasodilation was related to endothelial BK_Ca_ channel. Meanwhile, iberiotoxin significantly inhibited Jujuboside B-induced vasodilation, confirming that Jujuboside B reduced vascular tension involving hyperpolarization through opening endothelial BK_Ca_ channel. Moreover, blocking K_ATP_ channel with glibenclamide significantly attenuated Jujuboside B-induced vasorelaxation in endothelium-intact vascular rings, suggesting that Jujuboside B reduce vascular tension involving hyperpolarizaiton through opening K_ATP_ channel. However, glibenclamide did not affect SNP-induced vasodilation in endothelium-intact or endothelium-denuded vascular rings, suggesting that K_ATP_ channel of VSMCs was not involved in SNP-induced vasodilation. That is to say, Jujuboside B reduced vascular tension involving hyperpolarizaiton through opening endothelial K_ATP_ channel. Thus, we speculated that endothelial BK_Ca_ and K_ATP_ channel participated in Jujuboside B-induced vasodilation and hyperpolarized membrane after Jujuboside B induced Ca^2+^ influx and eNOS phosphorylation. However, the specific regulatory effect of Jujuboside B on potassium channels in vasculature and the underlying mechanisms needed to be researched further.

In conclusion, the present study proposed the novel pharmacological action of Jujuboside B on reducing vascular tension and protecting endothelial function. The potential mechanisms involved that Jujuboside B induced eNOS activation by increasing Ca^2+^ influx through endothelial TRPC channels and induced hyperpolarization by opening endothelial BK_Ca_ and K_ATP_ channels. The vascular dilative and protective effects of Jujuboside B were beneficial for treating endothelial dysfunction and vascular diseases.

## Supporting Information

S1 Fig Effect of PI_3_K, PKA and AMPK on Jujuboside B-induced vasodilation. (TIF)
