# Supplementary figures and images for: Jujuboside B Reduces Vascular Tension by Increasing Ca2+ Influx and Activating Endothelial Nitric Oxide Synthase
Source: PLoS One. 2016 Feb 22;11(2):e0149386. doi: 10.1371/journal.pone.0149386 (PMC4762982; doi:10.1371/journal.pone.0149386)

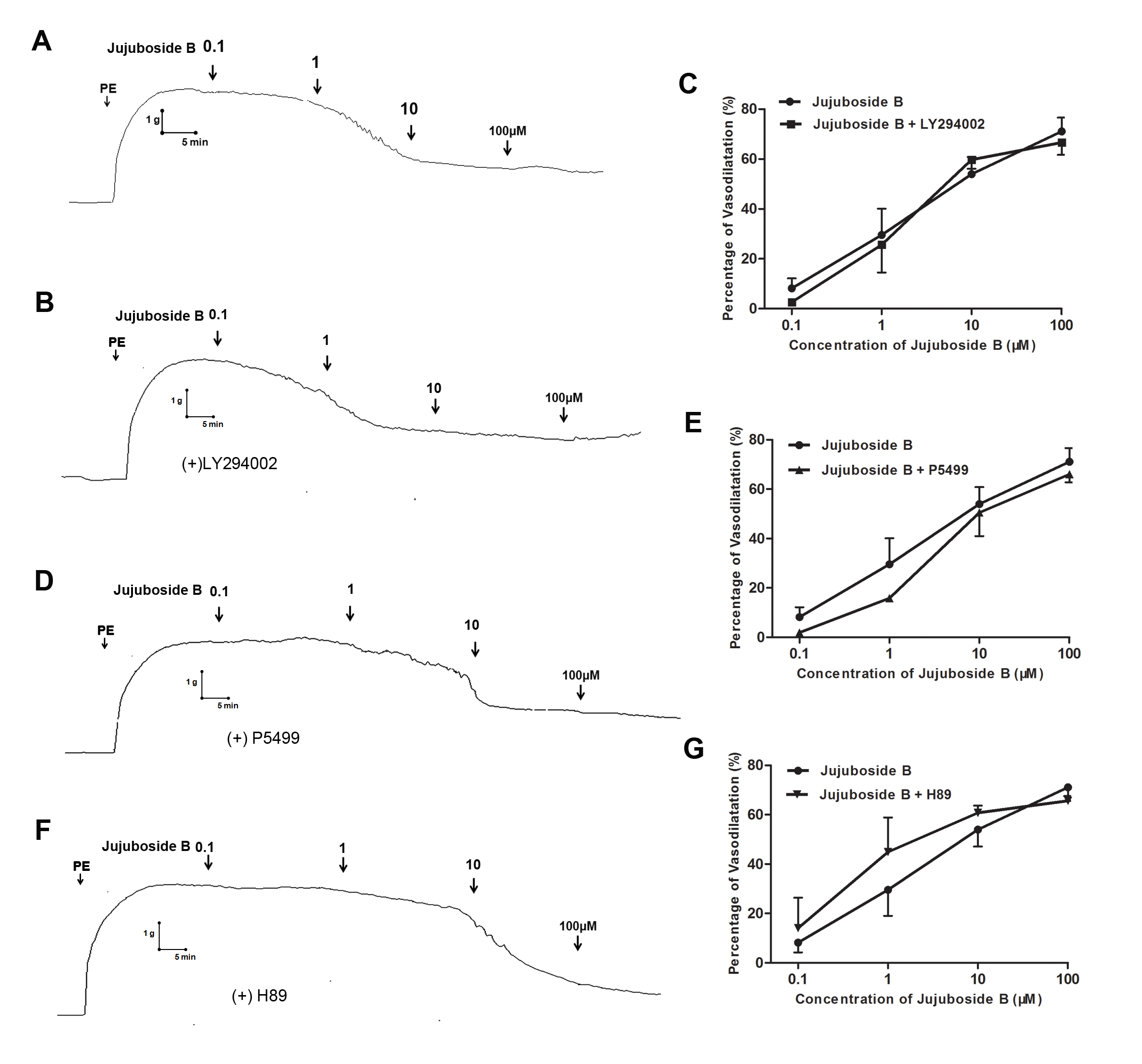

Supplement: S1 Fig — (TIF) [file pone.0149386.s001.tif]
